# Subjective and objective experiences of childhood adversity: a meta‐analysis of their agreement and relationships with psychopathology

**DOI:** 10.1111/jcpp.13803

**Published:** 2023-04-26

**Authors:** Emma R. Francis, Anna Tsaligopoulou, Sarah E. Stock, Jean‐Baptiste Pingault, Jessie R. Baldwin

**Affiliations:** ^1^ Division of Psychology and Language Sciences, Department of Clinical, Educational and Health Psychology University College London London UK; ^2^ Child Study Centre Yale University School of Medicine New Haven CT USA; ^3^ Institute of Epidemiology & Health University College London London UK; ^4^ Social, Genetic and Developmental Psychiatry Centre Institute of Psychiatry, Psychology and Neuroscience, King's College London London UK

**Keywords:** Childhood adversity, subjective measures, objective measures, psychopathology, meta‐analysis

## Abstract

**Background:**

Researchers use both subjective self‐report and objective measures, such as official records, to investigate the impact of childhood adversity on psychopathology. However, it is unclear whether subjective and objective measures of childhood adversity (a) show agreement, and (b) differentially predict psychopathology.

**Method:**

To address this, we conducted a pre‐registered meta‐analysis to examine the agreement between subjective and objective measures of childhood adversity, and their prediction of psychopathology. We searched in PubMed, PsycINFO and Embase for articles with both subjective measures (self‐reports) and objective measures of childhood adversity (comprising official records, or reports from multiple informants unrelated to the target individual), and measures of psychopathology.

**Results:**

We identified 22 studies (*n* = 18,163) with data on agreement between subjective and objective measures of childhood adversities, and 17 studies (*n* = 14,789) with data on the associations between subjective and objective measures with psychopathology. First, we found that subjective and objective measures of childhood adversities were only moderately correlated (e.g. for maltreatment, *r* = .32, 95% CI = 0.23–0.41). Second, subjective measures of childhood adversities were associated with psychopathology, independent of objective measures (e.g. for maltreatment, *r* = .16, 95% CI = 0.09–0.22). In contrast, objective measures of childhood adversities had null or minimal associations with psychopathology, independent of subjective measures (e.g. *r* for maltreatment = .06, 95% CI = −0.02–0.13).

**Conclusions:**

Our findings suggest that the effects of childhood adversity on psychopathology are primarily driven by a person's subjective experience. If this is the case, clinical interventions targeting memories and cognitive processes surrounding childhood adversity may reduce the risk of psychopathology in exposed individuals.

## Introduction

Childhood adversities, such as maltreatment, bullying and neighbourhood deprivation, are well‐established risk factors for psychopathology (Kessler, Davis, & Kendler, 1997). However, it is unclear if risk for psychopathology is driven by the subjective or objective experience of childhood adversity. Answering this question is critical to understand the pathways leading from childhood adversity to psychopathology, and, in turn, develop effective interventions.

Childhood adversity can be measured through a variety of different methods that index the subjective or objective experience. Most commonly, self‐reports are used that assess an individual's subjective appraisal and memory of their experiences. Less often, more objective measures are used that do not rely on the target individual's perception of their experiences, but rather legal definitions (e.g. crime records to assess violence exposure) (Goldman‐Mellor, Margerison‐Zilko, Allen, & Cerda, 2016), safeguarding concerns (e.g. child protection records to assess maltreatment) (Everson et al., 2008) or consensus across multiple informants unrelated to the target individual (e.g. peer nominations to assess bullying) (Kochel, Bagwell, Ladd, & Rudolph, 2017). Though both subjective and objective measures are used to study the consequences of childhood adversity, such measures may not capture the same individuals. For example, a meta‐analysis found that retrospective self‐reports of childhood maltreatment showed poor agreement with prospective measures, mainly based on more objective assessments such as official records, research worker observations and parent reports (Cohen's kappa = .19) (Baldwin, Reuben, Newbury, & Danese, 2019). This suggests that subjective and objective measures of childhood adversity might capture partially distinct groups of individuals.

If subjective and objective measures of childhood adversity do capture distinct groups of individuals, then both measures may be differentially associated with psychopathology. Indeed, initial evidence suggests that subjective measures of childhood adversity may show stronger associations with psychopathology than objective measures (Baldwin & Esposti, 2021). For example, one study found that subjective self‐reports of child maltreatment were associated with an increased risk of psychopathology in adulthood, independent of court‐documented evidence (Danese & Widom, 2020). However, in the absence of self‐reports, court records of maltreatment were not associated with psychopathology. This finding does not appear to be limited to studies examining child maltreatment, as similar findings have been found across other childhood adversities such as bullying victimisation (Bouman et al., 2012) and living in an area with neighbourhood disorder (Newbury et al., 2017). For example, one study observed that an increased risk of internalising problems was limited to subjective self‐reports of bullying victimisation rather than peer nominations (i.e. reports from multiple children in a classroom) (Bouman et al., 2012). In addition, perceptions of neighbourhood disorder are associated with elevated risk of psychotic experiences, after accounting for objective levels of crime and disorder (Newbury et al., 2017). However, despite such evidence from individual studies, there has been no systematic evaluation of the relative contributions of subjective and objective measures of childhood adversity to psychopathology. Determining whether subjective experiences of childhood adversity drive an increased risk of psychopathology is critical to inform clinical practice, as such findings would indicate that therapeutic approaches that address perceptions of adversity could reduce related psychopathology.

To address these research gaps, we conducted a pre‐registered meta‐analysis of studies with subjective and objective measures of childhood adversity and assessment of psychopathology. Our objectives were to examine (a) the agreement between subjective and objective measures of childhood adversity, (b) the independent contribution of subjective and objective measures of childhood adversity to psychopathology, and (c) moderators of these effects.

## Method

### Protocol and registration

This meta‐analysis was pre‐registered in the PROSPERO International prospective register of systematic reviews (CRD42021239454). In the pre‐registered protocol, our primary review question regarded the association between subjective and objective measures of childhood adversity and psychopathology; however, we specified that we could also assess meta‐analytic agreement between subjective and objective measures if sufficient data were available (which was the case). We conducted this meta‐analysis in line with PRISMA guidelines (Table S1).

### Inclusion criteria

Studies were eligible if they (a) included subjective and objective measures of childhood adversity and (b) had data on the relative associations between subjective and objective measures of childhood adversity with psychopathology, and/or the agreement between subjective and objective measures of childhood adversity. *Subjective measures* were defined as an individual's perception of their own adverse childhood experiences, captured through self‐reported interviews or questionnaires. These measures assessed whether an event occurred (e.g. maltreatment) rather than its subjective impact. *Objective measures* were defined as assessments unlikely to be affected by the target individual's perception of their experience, such as (a) official records (e.g. child protection records, crime records or medical records) or (b) reports derived from multiple individuals who are not directly related to the individual (e.g. peer nominations for bullying). Note that for (b), reports were required from multiple informants, rather than a single individual, to maximise accuracy. Psychopathology was defined as diagnoses or symptoms of a psychiatric illness. We excluded studies that included non‐human animals or human participants from a selected clinical sample or a clinical trial.

### Literature search

We searched Embase, MEDLINE and PsycINFO using the Ovid platform for peer‐reviewed articles written in English and published from database inception to March 2021. Search terms are shown in Appendix S1. We included general search terms indexing child adversity and trauma, as well as specific terms indexing adversities known to have been previously assessed with both subjective and objective measures (e.g. maltreatment, bullying and neighbourhood adversities). Additional studies were identified via searching reference lists of included studies.

### Study selection

Two authors (E.R.F. and S.E.S.) independently screened abstracts and titles before reviewing the full text of potentially eligible articles. Uncertainty of study inclusion was resolved through discussion with a third reviewer (J.R.B.).

### Data extraction

Data on sample characteristics and effect sizes for the relative associations between subjective and objective measures of childhood adversity and psychopathology were systematically extracted from each article by two independent reviewers (E.R.F. and A.T.), blind to the other's data extraction (details in Appendix S2). For data on the agreement between subjective and objective measures of adversity, one author (J.R.B.) extracted or calculated effect sizes from available data (correlations and/or Cohen's kappas), and this information was checked by another author (E.R.F.). Relevant missing information was requested from study authors.

We extracted information on study quality (risk of bias) for each article using an adapted version of the Newcastle‐Ottawa Scale (Wells et al., 2000), shown in Table S2. All articles were independently assessed by three reviewers (E.R.F., A.T. and J.R.B.). Results for each study are shown in Table S3.

### Effect size conversion

#### Effect size for the agreement between subjective and objective measures of childhood adversity

Studies with data on the agreement between subjective and objective measures of childhood adversity reported either (a) data to derive a contingency table comparing binary subjective measures of childhood adversity (yes/no) with binary objective measures of childhood adversity (yes/no) or (b) a Pearson's correlation coefficient for the association between continuous subjective and objective measures of childhood adversity. To derive a common effect size metric (namely, a correlation coefficient), we used data in the contingency tables to calculate tetrachoric correlations, which are directly comparable to the Pearson's correlations reported by many of the studies.

#### Effect sizes for the associations between subjective and objective measures of childhood adversity and psychopathology

We converted effect sizes for the independent associations between subjective and objective measures of childhood adversity and psychopathology to partial correlation coefficients (*r*). Partial correlation coefficients represent the association between subjective measures of childhood adversity and psychopathology, controlling for objective measures of adversity, and vice versa. Formulae for converting effect sizes are shown in Table S4. Where studies reported bivariate correlations between (a) subjective and objective measures with psychopathology and (b) subjective and objective measures, we calculated partial correlations using a procedure described in Appendix S3.

### Data analysis

Analyses were performed using the ‘metafor’ package (Viechtbauer, 2010) in R (version 4.1.2). First, we examined the agreement between subjective and objective measures of childhood adversity. To do so, we conducted separate random‐effects multi‐level meta‐analysis models to pool the agreement (*r*) between subjective and objective measures of childhood adversity, with different models for different adversity types. To account for interdependencies between multiple effect sizes from single studies and samples (Assink & Wibbelink, 2016), four different sources of variance were modelled: (1) sample variance of the effect sizes, (2) variance between effect sizes extracted from the same study, (3) variance between studies and (4) variance between samples (for instances where the same sample was used across multiple studies). To stabilise the variances, we transformed correlations to Fisher's *z* prior to meta‐analysis (Borenstein, Hedges, Higgins, & Rothstein, 2021) and back‐transformed the meta‐analytic results to *r* using Fisher's *z*‐to‐*r* transformation for interpretability (Borenstein et al., 2021). For the meta‐analysis of agreement between subjective and objective measures of child maltreatment, Cohen's kappa effect sizes were available (as well as correlation coefficients), and so we also conducted a random‐effects multi‐level meta‐analysis model for the agreement measured via Cohen's kappa. We then conducted a post‐hoc analysis to examine whether the agreement between subjective and objective measures was moderated by the type of childhood adversity.

Second, for each form of childhood adversity, we tested the meta‐analytic association (a) between subjective measures and psychopathology, controlling for corresponding objective measures, and (b) between objective measures and psychopathology, controlling for subjective measures. To do so, we used the same meta‐analytic procedure as specified above (i.e. random‐effects multi‐level meta‐analysis models with three sources of variance, using the Fisher's z transformation and z‐to‐r back‐transformation).

Third, we examined whether the independent associations between subjective and objective measures of childhood adversity were moderated by various predictors. Where sufficient data were available, we used meta‐regression to test whether heterogeneity in effect sizes was predicted by the informant reporting on psychopathology (self vs. other), type of study (longitudinal vs. cross‐sectional assessment of childhood adversity and psychopathology), type of psychopathology outcome (internalising or externalising problems), sex distribution of the sample and study quality.

### Risk of bias across studies

We assessed the risk of bias across studies in two ways. First, we carried out a test for publication bias by performing an Egger's regression test for each multi‐level random‐effects meta‐analysis (Egger, Smith, Schneider, & Minder, 1997). Second, we performed leave‐one‐out analysis for each meta‐analysis, which assessed the undue effect of each individual study by testing changes in the meta‐analytic effect estimates when each study was omitted in turn.

### Data and code availability

The dataset and analysis code are available at: https://github.com/erfrancis/MetaAnalysis_ObjectiveSubjective.

## Results

### Search results

The study selection procedure is shown in Figure S1. We identified 22 studies with data on the agreement between subjective and objective measures of childhood adversity (see Table 1 for study details). These studies were based on 21 cohorts including 18,163 independent participants (51.3% female), with an average age of 14.8 years. As shown in Table 1, 9 studies focused on child maltreatment (41 effect sizes), 11 studies focused on bullying victimisation (20 effect sizes) and 2 studies focused on neighbourhood adversity (2 effect sizes). There were more effect sizes than studies as individual studies often reported multiple effect sizes. The average study quality score was 4 (range = 2–6), from a possible range of 0 (indicating very high bias) to 8 (indicating very low bias) using the adapted NOS (Table S3).

**Table 1:** Summary of study characteristics included in the meta‐analysis for subjective and objective measures of childhood adversity

Author (year)	Country	Total analytical sample size (% female)	Exposure type (objective)	Exposure type (subjective)	Type of objective measure	Type of subjective measure	Type of mental health outcome(s)	Average age of self‐reported adversity (years)	Informant for psychopathology	Included in meta‐analysis on agreement (Y/N)	Included in meta‐analysis on psychopathology (Y/N)
*Child maltreatment*											
Cho and Jackson (2016)	U.S.A	285 (45.1)	Child maltreatment: Sexual abuse, physical abuse, emotional abuse	Child maltreatment: Sexual abuse, physical abuse, emotional abuse	Child Protection Service Records	Self‐report interview	Internalising symptoms, Externalising symptoms	13.3	Parent	Y	Y
Danese and Widom (2020)	U.S.A.	1,196 (48.7)	Child maltreatment	Child maltreatment	Crime records	Self‐report interview	Any psychopathology diagnosis, Any internalising disorder diagnosis, Any externalising disorder diagnosis, Depression diagnosis, Dysthymia diagnosis, Generalised anxiety disorder diagnosis, PTSD diagnosis, Antisocial personality disorder diagnosis, Alcohol abuse and/or dependence diagnosis, Drug abuse and/or dependence	28.7	Self‐report	Y	Y
Everson et al. (2008)	U.S.A	350 (51)	Child maltreatment: Physical abuse, sexual abuse, psychological abuse	Child maltreatment: Physical abuse, sexual abuse, psychological abuse	Child Protection Service Records	Self‐report questionnaire	Psychological adjustment symptoms	12	Self‐report	Y	Y
Havlicek and Courtney (2016)	U.S.A	474 (Wave 1: 53 and Wave 2: 56)	Child maltreatment, Physical abuse, sexual abuse, neglect	Child maltreatment, Physical abuse, sexual abuse, neglect	Child Protection Service Records	Self‐report interview	N/A	Wave 1: 17.5 and Wave 2: 19	N/A	Y	N^a^
McGee, Wolfe, Yuen, Wilson, and Carnochan (1995)	Canada	160 (56.3)	Child maltreatment: Physical violence, family violence, sexual abuse, emotional abuse, neglect	Child maltreatment: Physical violence, family violence, sexual abuse, emotional abuse, neglect	Child Protection Service Records	Self‐report interview	Internalising symptoms, Externalising symptoms	13.8	Self‐report	Y	Y
Negriff, Schneiderman, and Trickett (2017)	U.S.A	221 (50)	Child maltreatment: Sexual abuse, physical abuse, emotional abuse, neglect	Child maltreatment: Sexual abuse, physical abuse, emotional abuse, neglect	Child Protection Service Records	Self‐report interview	Depression symptoms, PTSD symptoms, Anxiety symptoms, Marijuana Use, Alcohol use, Person offences externalising problems, Property offences externalising problems	18.49	Self‐report	Y	Y
Sierau et al. (2017)	Germany	944 (47.2)	Child maltreatment: Failure to provide, Lack of supervision, Physical abuse, Emotional maltreatment	Child maltreatment: Failure to provide, Lack of supervision, Physical abuse, Emotional maltreatment	Child Protection Service Records	Self‐report interview	N/A	10.1	N/A	Y	N^a^
Smith et al. (2008)	U.S.A	1,000 (50)	Child maltreatment	Child maltreatment	Child Protection Service Records	Self‐report interview	N/A	23	N/A	Y	N^a^
White et al. (2016)	U.S.A	770 (54.8)	Emotional maltreatment including violation of psychological safety and security; failure to support acceptance and self‐esteem; failure to allow age‐appropriate autonomy; and restriction (e.g. confinement/isolation, binding)	Emotional maltreatment including violation of psychological safety and security; failure to support acceptance and self‐esteem; failure to allow age‐appropriate autonomy; and restriction (e.g. confinement/isolation, binding)	Child Protection Service Records	Self‐report interview	Anxiety symptoms, Depression symptoms, Suicidal symptoms	14	Self‐report	Y	Y
*Bullying victimisation*											
Bouman et al. (2012)	Netherlands	1,192 (49.8)	Bullying victimisation	Bullying victimisation	Peer nomination	Self‐report interview	Depression symptoms, anxiety symptoms	11.2	Self‐report	Y	Y
De Los Reyes and Prinstein (2004)	U.S.A	203 (60)	Bullying victimisation	Bullying victimisation	Peer nomination	Self‐report questionnaire	N/A	16.31	N/A	Y	N^a^
Flanagan et al. (2008)	U.S.A	383 (57)	Bullying victimisation	Bullying victimisation	Peer nomination	Self‐report questionnaire	Social anxiety symptoms	12.8	Parent	Y	Y
Graham et al. (2003)	U.S.A	785 (55.7)	Bullying victimisation	Bullying victimisation	Peer nomination	Self‐report questionnaire	Anxiety symptoms, depression symptoms, internalising symptoms, externalising symptoms	11.5	Self‐report and teacher	Y	Y
Graham and Juvonen (1998)	U.S.A	418 (50.7)	Bullying victimisation	Bullying victimisation	Peer nomination	Self‐report questionnaire	Social anxiety symptoms	12.4	Self‐report	Y	Y
Gromann et al. (2013)	Netherlands	724 (48.3)	Bullying victimisation	Bullying victimisation	Peer nomination	Self‐report questionnaire	Non‐clinical psychotic experiences symptoms	11.9	Self‐report	Y	Y
Kochel et al. (2017)	U.S.A	5th, 6th grade: 483; 9th, 10th grade: 444 (49.69)	Peer victimisation	Peer victimisation	Peer nomination	Self‐report questionnaire	Depressive symptoms	13.45	Self‐report, teacher and parent	Y	Y
McClain et al. (2020)	U.S.A	Male: 212 Female: 270 (56)	Overt victimisation, relational victimisation	Bullying victimisation	Peer nomination	Self‐report questionnaire	Depressive symptoms, anxiety symptoms	9.16	Self‐report	Y	Y
Mulder et al. (2017)	Netherlands	Time 1: 1100 Time 2: 1139 (46)	Bullying victimisation	Bullying victimisation	Peer nomination	Self‐report questionnaire	Social anxiety symptoms	12	Self‐report	Y	Y
Rigby and Slee (1999)	Australia	450 and 395 (46.7)	Bullying victimisation	Bullying victimisation	Peer nomination	Self‐report questionnaire	N/A	15	N/A	Y	N^a^
Zimmer‐Gembeck and Pronk (2012)	Australia	335 (52.8)	Bullying victimisation	Bullying victimisation	Peer nomination	Self‐report questionnaire	Depressive symptoms, social anxiety symptoms	12.5	Self‐report	Y	Y
*Neighbourhood adversity*											
Newbury et al. (2017)	U.K.	2066 (51)	Neighbourhood crime	Neighbourhood disorder	Crime records	Self‐report interview/questionnaire	Psychotic experience symptoms	18.4	Self‐report	Y	Y
Goldman‐Mellor et al. (2016)	U.S.A	4,462 (49.2)	Neighbourhood violence	Neighbourhood safety	Crime records	Self‐report questionnaire	Serious psychological distress symptoms	14.65	Self‐report	Y	Y

^a^ Five studies included in the meta‐analysis of agreement between subjective and objective measures were not included in the meta‐analysis on the independent associations between these measures and psychopathology (De Los Reyes & Prinstein, 2004; Havlicek & Courtney, 2016; Rigby & Slee, 1999; Sierau et al., 2017; Smith et al., 2008). This was due to these studies not containing effect sizes that could be extracted or calculated from the data available, or no contact from study author.

We identified 17 studies with data on the independent associations between both subjective and objective measures of childhood adversity and psychopathology (see Table 1 for study details). These 17 studies were based on 15 cohorts comprising 14,789 independent participants (54.1% female) with an average age of 14.3 years. Among these studies, 6 focused on maltreatment (188 effect sizes), 9 focused on bullying victimisation (90 effect sizes) and 2 focused on neighbourhood adversity (4 effect sizes).

### What is the agreement between subjective and objective measures of childhood adversity?

#### Child maltreatment

**Figure 1:**
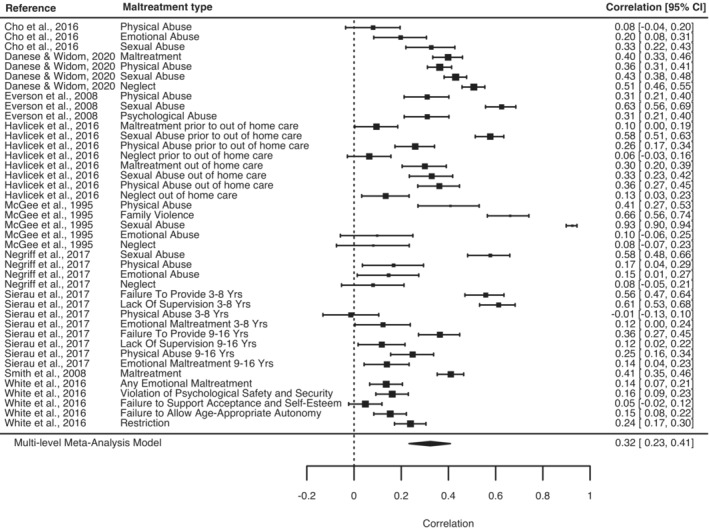
Forest plot for studies examining the correlation between subjective and objective measures of childhood maltreatment

We first examined the meta‐analytic agreement between subjective self‐reports of childhood maltreatment and objective measures (comprising child protection records or court records). The correlation between subjective and objective measures of maltreatment was only medium in magnitude (*r* = .32, 95% CI, 0.23–0.41; *p* < .0001; 41 effect sizes) (Figure 1). Furthermore, the agreement between subjective and objective measures of maltreatment as assessed through Cohen's kappa was poor (*k* = .16, 95% CI, 0.10–0.22; *p* < .0001), indicating that agreement was only 16% greater than that expected due to chance. We did not find evidence of publication bias, and leave‐one‐out analysis suggested that the meta‐analytic estimates were not unduly influenced by any individual study (Table S5).

#### Bullying victimisation

**Figure 2:**
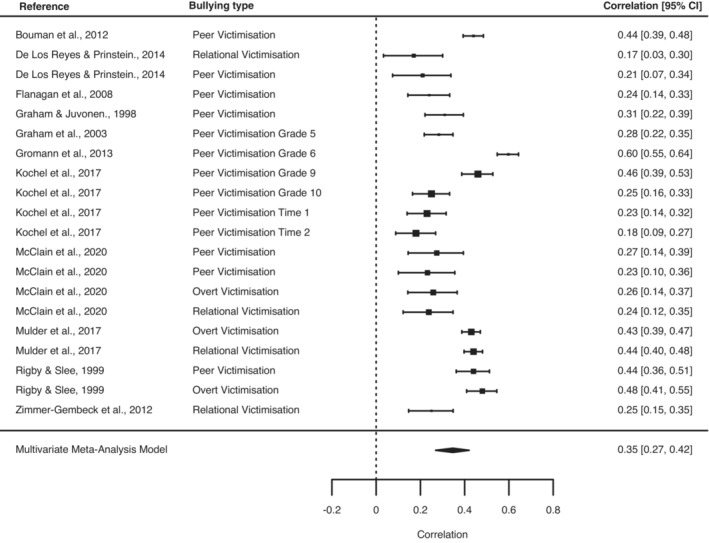
Forest plot for studies examining the correlation between subjective and objective measures of bullying victimisation

We next examined the agreement between subjective self‐reports of bullying victimisation and objective measures (comprising peer nominations from multiple children in a classroom). The correlation between subjective and objective measures of bullying victimisation was medium in magnitude (*r* = .35, 95% CI, 0.27–0.42; *p* < .0001, 20 effect sizes) (Figure 2). The Egger's test was statistically significant (Q_moderation = 7.8016; *p* = .0052) but visual inspection of the effect sizes showed that smaller studies reported smaller, rather than larger effect sizes which would be indicative of publication bias (Figure S2). Leave‐one‐out analysis suggested that the meta‐analytic estimate was not unduly influenced by any individual study (Table S5).

#### Neighbourhood adversity

We then examined the agreement between subjective self‐reports of neighbourhood violence/disorder and corresponding objective measures (crime records). Notably, only two studies contained available data, so these findings should be interpreted as preliminary. The correlation between the subjective and objective measures of neighbourhood adversity was small to medium in magnitude (*r* = .25, 95% CI, 0.11–0.39; *p* = .0007, 2 effect sizes) (Figure S3). We did not conduct an Egger's test or leave‐one‐out analysis due to the limited number of studies.

### Is the agreement between subjective and objective measures moderated by the type of childhood adversity?

We next conducted a post‐hoc (i.e. non‐pre‐registered) analysis to examine whether heterogeneity in the agreement between subjective and objective measures was influenced by the type of childhood adversity. We found that agreement between subjective and objective measures differed according to the type of childhood adversity (Q_moderation = 25.28, *p* = .0003), with stronger agreement for sexual abuse (*r* = .60, CI, 0.48–0.69; *k* [number of studies] = 6, ES [number of effect sizes] = 7) than for other forms of adversity, including physical abuse (*r* = .25, CI, 0.11–0.38; *k* = 7, ES = 9), emotional abuse (*r* = .21, CI, 0.07–0.33; *k* = 6, ES = 12), neglect (*r* = .30, CI, 0.16–0.43; *k* = 5, ES = 9), broad measures of maltreatment (*r* = .33, CI, 0.13–0.50; *k* = 3, ES = 4), bullying victimisation (*r* = .33, CI, 0.24–0.42; *k* = 11, ES = 20) and neighbourhood adversity (*r* = .25, CI, −0.03–0.50; *k* = 2, ES = 2).

### Do subjective and objective measures of childhood adversity independently predict psychopathology?

#### Child maltreatment

**Figure 3:**
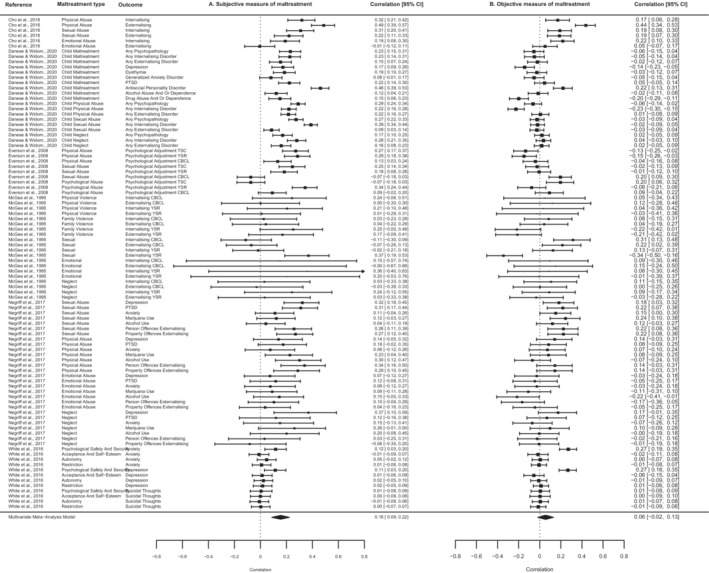
Meta‐analytic associations between subjective measures of child maltreatment and psychopathology, independent of objective measures (Panel A), and objective measures of child maltreatment and psychopathology, independent of subjective measures (Panel B)

Next, we examined the relative associations between subjective and objective measures of childhood maltreatment and psychopathology. Subjective self‐reports of maltreatment were significantly associated with psychopathology, independent of objective measures (*r* = .16, 95% CI, 0.09–0.22; *p* < .0001; 90 effect sizes; Figure 3). In contrast, objective measures of maltreatment were not associated with psychopathology, independent of subjective measures (*r* = .06, 95% CI, −0.02–0.13; *p* = .14; 90 effect sizes; Figure 3). We did not find evidence of publication bias for either meta‐analysis, and findings were not unduly influenced by any individual study (Table S6).

#### Bullying victimisation

**Figure 4:**
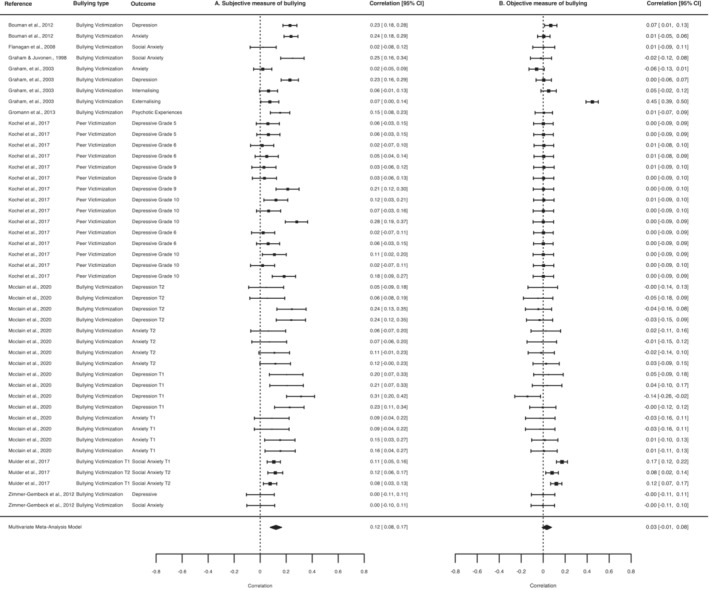
Meta‐analytic associations between subjective measures of bullying victimisation and psychopathology, independent of objective measures (Panel A), and objective measures of bullying victimisation and psychopathology, independent of subjective measures (Panel B)

Similar to the findings on child maltreatment, subjective self‐reports of bullying victimisation were significantly associated with psychopathology, independent of objective measures (*r* = .12, 95% CI, 0.08–0.17; *p* < .0001; 45 effect sizes; Figure 4). However, objective measures of bullying victimisation were not significantly associated with psychopathology, independent of subjective measures (*r* = .03, 95% CI, −0.01–0.08; *p* = .13; 45 effect sizes; Figure 4). We did not find evidence of publication bias for either meta‐analysis, although smaller studies were more likely to report smaller independent effects of objective measures on psychopathology (Figure S4). Findings for both meta‐analyses were not unduly influenced by any individual study (Table S6).

#### Neighbourhood adversity

Among the two available studies, subjective self‐reports of neighbourhood adversity were significantly associated with psychopathology, independent of objective measures (*r* = .26, 95% CI, 0.22–0.29; *p* < .001; 2 effect sizes; Figure S5). Objective measures of neighbourhood adversity also showed a small association with psychopathology, independent of subjective measures (*r* = .04, 95% CI, 0.02–0.07; *p* = .0003; 2 effect sizes; Figure S5), although this effect size was significantly smaller than the association between subjective measures and psychopathology (*p* < .001). Given the small number of studies with data for neighbourhood adversity, we did not conduct further analyses assessing publication bias, undue influence of individual studies and moderation of these effects.

### What moderates the independent associations between subjective and objective measures of childhood adversity and psychopathology?

Lastly, we examined predictors of heterogeneity in the relative associations between subjective and objective measures of childhood adversity with psychopathology. As the meta‐analytic findings were very similar for maltreatment and bullying, we combined effect sizes for both exposures in the moderation analyses to benefit from greater statistical power (Table 2). However, we present results for maltreatment and bullying separately in Table S7 for transparency.

**Table 2:** Moderators of the association between subjective and objective measures of bullying victimisation and childhood maltreatment, and psychopathology

Moderators by child adversity measure	No. of studies	No. of effect sizes	Effect size estimate, *r* (95% CI)	*Q* moderation	*p* value^a^
Subjective measure of child adversity					
Informant for psychopathology					
Self‐report	13	118	0.15 (0.11–0.20)	4.87	**.027**
Other informant	4	21	0.07 (0.00–0.15)		
Study type					
Cross‐sectional	14	113	0.15 (0.11–0.19)	4.11	**.043**
Longitudinal	4	26	0.09 (0.03–0.15)		
Type of psychopathology					
Internalising problems	13	88	0.14 (0.09–0.18)	0.04	.84
Externalising problems	5	37	0.13 (0.07–0.19)		
Sex (percentage female)	15	139	−0.00 (−0.00–0.00)	0.12	.73
Study quality	15	139	−0.00 (−0.04–0.04)	0.00	.99
Objective measure of child adversity					
Informant for psychopathology					
Self‐report	13	118	0.02 (−0.02–0.06)	6.40	**.011**
Other informant	4	21	0.11 (0.05–0.18)		
Study type					
Cross‐sectional	14	113	0.04 (0.00–0.08)	0.06	.81
Longitudinal	4	26	0.03 (−0.03–0.10)		
Type of psychopathology					
Internalising problems	13	88	0.04 (0.00–0.08)	1.29	.26
Externalising problems	5	37	0.07 (0.01–0.13)		
Sex (percentage female)	15	139	−0.00 (−0.00–0.00)	1.34	.25
Study quality	15	139	−0.02 (−0.06–0.02)	0.74	.39

^a^ The bold *p* values represent significance at <.05.

First, we found that the independent association between subjective measures of childhood adversity and psychopathology was stronger when psychopathology was self‐reported (*r* = .15, 95% CI = 0.11–0.20) versus reported by another informant (i.e. a parent or teacher, *r* = .07, 95% CI = 0.00–0.15, Q_moderation = 4.87, *p* = .027). In contrast, objective measures of childhood adversity showed a stronger independent association with psychopathology reported by another informant (*r* = .11, 95% CI = 0.05–0.18) versus self‐reports (*r* = .02, 95% CI = −0.02–0.06, Q_moderation = 6.40, *p* = .011).

Second, the independent association between subjective measures of childhood adversity and psychopathology was stronger when psychopathology was assessed concurrently to self‐reports of adversity (*r* = .15, 95% CI = 0.11–0.19) versus longitudinally (*r* = .09, 95% CI = 0.03–0.15, Q_moderation = 4.11, *p* = .043). No such moderation effect was found for objective measures of childhood adversity (Table 2).

Finally, the independent associations between subjective and objective measures of childhood adversity with psychopathology were not moderated by the type of psychopathology, sex distribution of the sample or study quality (Table 2).

The findings were broadly similar when maltreatment and bullying were examined separately (Table S7), although there was no statistically significant moderation by informant for analyses on maltreatment, or study type for analyses on bullying (though the direction of effects was consistent).

## Discussion

This meta‐analysis examined whether subjective and objective measures of childhood adversity overlap, and are independently associated with psychopathology. First, we found only modest associations between subjective and objective measures of childhood adversity. Second, we found that subjective measures of childhood adversity were associated with psychopathology, independent of corresponding objective measures. In contrast, objective measures of childhood adversity had null or very small associations with psychopathology, independent of subjective measures. These findings were consistent across multiple types of childhood adversity, including childhood maltreatment, bullying victimisation and neighbourhood violence, relying on different types of objective measures (e.g. child protection records, peer nominations and crime records).

The modest associations between subjective and objective measures of childhood adversity suggest that individual perceptions and memories of adverse experiences do not closely match what is recorded more objectively (such as through child protection or crime records, or reports across multiple informants). These findings mirror low agreement observed between retrospective self‐reports of child maltreatment with prospective measures (assessed through parent/informant reports as well as official records) (Baldwin et al., 2019), as well as between self‐reports and objective records of other experiences, such as media use (Parry et al., 2021).

There are several plausible explanations for moderate agreement between subjective and objective measures of childhood adversity. On the one hand, objective measures might identify only the most severe or visible cases of childhood adversity (such as from official records or peer nominations from multiple informants), and self‐report measures may detect more true cases (Mulder, Hutteman, & van Aken, 2017). On the other hand, subjective measures might be less accurate in detecting childhood adversity because of biases due to individual motivations and memory (Baldwin et al., 2019). For example, some may underreport or minimise adversity experienced due to social desirability bias (Fisher, Bunn, Jacobs, Moran, & Bifulco, 2011), self‐protective mechanisms, personality traits (e.g. high agreeableness; Reuben et al., 2016) or fear of perpetrator repercussions. Various memory fallibilities can also limit accuracy of self‐reports, such as not remembering adversity in early childhood due to infantile amnesia (Lebois et al., 2021), or over‐recalling adversity due to a negative bias in autobiographical memory involved in psychopathology (Colman et al., 2016). Notably though, the majority of studies included in our meta‐analysis involved self‐reports obtained prospectively in childhood (Table 1), which reduces the likelihood that the results we found are due to inaccuracies in retrospective memory. Finally, it is possible that low agreement was due to differences in the assessment of childhood adversity (e.g. in the definition used, or observational period assessed) between subjective and objective measures (Baldwin et al., 2019). However, only a few studies reported different definitions of adversity (Goldman‐Mellor et al., 2016; McClain, Younginer, & Elledge, 2020; Newbury et al., 2017) or different observation periods (Gromann, Goossens, Olthof, Pronk, & Krabbendam, 2013; Newbury et al., 2017; Smith, Ireland, Thornberry, & Elwyn, 2008) between subjective and objective measures, and so low overall agreement cannot solely be due to these differences.

Notably, in post‐hoc analyses, we found that agreement between subjective and objective measures differed according to the type of childhood adversity. Specifically, there was higher agreement between subjective and objective measures of sexual abuse compared to physical and emotional abuse, neglect, and broader measures of maltreatment, bullying and neighbourhood adversity. This may be because sexual abuse is a more clear‐cut form of adversity compared to other experiences (e.g. emotional abuse or neglect, or bullying), which can involve a more subjective interpretation. Indeed, previous research showed higher agreement between prospective and retrospective measures of other clear‐cut forms of adversity (e.g. parental loss; Reuben et al., 2016), or childhood experiences (e.g. residence changes; Henry, Moffitt, Caspi, Langley, & Silva, 1994) compared to more ambiguous psychosocial experiences.

Our finding that subjective self‐reports of childhood adversity are more strongly associated with psychopathology than objective measures might be due to aetiological mechanisms or bias. With regard to aetiological mechanisms, perception and memories of adverse childhood experiences might mediate the relationship between objective experiences and mental ill health (Elwyn & Smith, 2013). That is, memories and recollections of traumatic experiences may drive the risk of psychopathology in those exposed to adversity, for example by evoking negative views about the self and others. In contrast, individuals exposed to childhood adversity who do not remember it, or do not perceive it to be adversity, may not develop psychopathology. This mediation interpretation is supported by the evidence that objective measures of childhood adversity are associated with psychopathology when subjective measures are not controlled for (Bouman et al., 2012; Cutajar et al., 2010; Kochel et al., 2017; Mills, Kisely, Alati, Strathearn, & Najman, 2016; Smith et al., 2008; Widom, DuMont, & Czaja, 2007), including in studies applying stringent causal inference methods (Capusan et al., 2021; Kugler et al., 2019).

With regard to bias, three potential explanations exist. First, the stronger association between subjective measures of childhood adversity and psychopathology relative to objective measures might partly be explained by reverse causation or recall bias. For example, individuals with mental ill health might be more likely to perceive current experiences as harmful due to cognitive biases (e.g. negative attentional bias) (Beck, 2008), or recall past experiences more negatively due to mood‐congruent memory (Brewin, Andrews, & Gotlib, 1993). Indeed, longitudinal research has suggested that increases in depression symptoms over time predicted small increases in retrospective reports of child maltreatment (Goltermann et al., 2021). We found some evidence to suggest the existence of such recall bias, as self‐reports of childhood adversity were more strongly associated with psychopathology in cross‐sectional studies than in longitudinal studies, suggesting that perceived childhood adversity is more closely related to concurrent than later mental ill health. However, it is also possible that such effect size differences might be due to effects of perceived childhood adversity on psychopathology decreasing over time.

Second, subjective measures of childhood adversities might be more strongly associated with psychopathology than objective measures due to shared method variance as self‐reports were used to assess subjective experiences and, in most instances, psychopathology (Widom & Shepard, 1996). In contrast, objective measures did not rely on self‐reports and showed minimal (or no) independent association with self‐reported psychopathology. This explanation is supported by our finding showing that self‐reports of childhood adversity were associated with psychopathology that was self‐reported, but not reported by another informant (though this appeared to be driven by studies on bullying). Similarly, previous studies found that retrospective self‐report measures of childhood adversity were associated with poor self‐reported outcomes relating to mental and physical health, but not objectively recorded outcomes (Gehred et al., 2021; Osborn & Widom, 2020; Reuben et al., 2016).

Third, the stronger relationship between subjective compared to objective measures of childhood adversity and psychopathology may partly be explained by a confounding variable that results in an individual perceiving experiences as more negative and also developing psychopathology. For example, previous research showed that personality traits such as neuroticism and low agreeableness are associated with recalling more childhood adversity than was recorded prospectively (Reuben et al., 2016), and these traits also predispose to psychopathology (Hengartner, Ajdacic‐Gross, Wyss, Angst, & Rössler, 2016). Furthermore, previous research found that genetic liability to psychopathology (e.g. depression and low well‐being) is associated with self‐reports of bullying victimisation, but not more objective measures (teacher or peer reports) (Armitage et al., 2022), implying potential for genetic confounding.

These findings should be interpreted in the context of some limitations. First, only two studies (Goldman‐Mellor et al., 2016; Newbury et al., 2017) focused on neighbourhood adversities, which limits our ability to draw conclusions for this form of childhood adversity. Nevertheless, we observed broadly similar findings to those observed for maltreatment and bullying victimisation. Second, the comparatively small number of studies included in each level of the moderator analyses prevents us from drawing strong conclusions about these results. For example, only four studies were longitudinal (Kochel et al., 2017; McClain et al., 2020; Mulder et al., 2017; White, English, Thompson, & Roberts, 2016), and only four studies assessed psychopathology through reports from other informants (rather than self‐reports) (Cho & Jackson, 2016; Flanagan, Erath, & Bierman, 2008; Graham, Bellmore, & Juvonen, 2003; Kochel et al., 2017). Third, because data were unavailable, we could not examine whether the findings were moderated by key factors, such as the time interval between childhood adversity exposure and psychopathology, and race or ethnicity. Finally, it is possible that measures of childhood adversity defined as ‘objective’ (e.g. official records and peer nominations) could still be partly influenced by the target individual's perceptions of their experiences (e.g. if children seek out support from official services or confide in their peers). Nevertheless, because official records and peer nominations rely on evidence from a large number of sources, they are likely to capture much more information than only the individual's subjective experience.

Our findings have implications for future research. To understand why subjective measures of childhood adversity are more strongly associated with psychopathology than objective measures, future studies should aim to test whether the finding is due to sources of bias or an aetiological mechanism. To understand the direction of the relationship (and test recall bias/reverse causation), longitudinal analyses are needed on datasets with repeated measures of self‐reported childhood adversity and psychopathology. To test shared method variance, studies could further examine whether subjective measures of childhood adversity are associated with psychopathology outcomes reported by multiple informants or through objective records. To test confounding, studies should examine the extent to which factors predisposing individuals to negative perceptions and psychopathology (e.g. personality traits, genetic vulnerabilities) account for the relationship between the subjective experience of adversity and psychopathology (Pingault et al., 2022).

If subjective appraisal of childhood adversity directly contributes to psychopathology, then therapeutic approaches which target perceptions and memories could help to reduce and prevent psychopathology (Danese & Widom, 2021). Such interventions might involve techniques that help to modify cognitive appraisal of the experience, the affective response and associated views about the self and others (Creamer, McFarlane, & Burgess, 2005). Of note, this finding would not undermine the importance of preventing objective experiences of childhood adversity, which is a moral priority for parents and society. Rather, it would provide new avenues for transdiagnostic cognitive interventions to protect survivors of childhood adversity from mental illness.

## Supporting information


**Appendix S1.** Search terms to identify eligible studies.
**Appendix S2.** Variables extracted.
**Appendix S3.** Converting unadjusted correlations to partial correlations.
**Table S1.** PRISMA checklist.
**Table S2.** The description of bias assessment.
**Table S3.** Risk of bias for all included studies (agreement and main meta‐analysis).
**Table S5.** Egger's test and leave‐one‐out analysis for studies examining the agreement between subjective and objective measures of childhood adversity.
**Table S6.** Egger's test and leave‐one‐out analysis for studies assessing whether subjective and objective measures of childhood adversity independently predict psychopathology.
**Table S7.** Moderators of the association between subjective and objective measures of adverse childhood experiences and psychopathology.
**Figure S1.** PRISMA flow diagram for the study inclusion process.
**Figure S2.** Correlation between subjective and objective measures of bullying victimisation according to study sample size.
**Figure S3.** Forest plot for studies examining the correlation between subjective and objective measures of neighbourhood adversity.
**Figure S4.** Correlation between objective measures of bullying victimisation with psychopathology independent of subjective measures, by study sample size.
**Figure S5.** Forest plot showing the meta‐analytic associations between subjective measures of neighbourhood adversity and psychopathology, independent of objective measures (panel A), and objective measures of neighbourhood adversity and psychopathology, independent of subjective measures (panel B).
