# Supplementary material for: Subjective and objective experiences of childhood adversity: a meta‐analysis of their agreement and relationships with psychopathology
Source: J Child Psychol Psychiatry. 2023 Apr 26;64(8):1185–99. doi: 10.1111/jcpp.13803 (PMC10617978; doi:10.1111/jcpp.13803)
Supplement: Supplementary file 1 — Appendix S1. Search terms to identify eligible studies. Appendix S2. Variables extracted. Appendix S3. Converting unadjusted correlations to partial correlations. Table S1. PRISMA checklist. Table S2. The description of bias assessment. Table S3. Risk of bias for all included studies (agreement and main meta‐analysis). Table S5. Egger's test and leave‐one‐out analysis for studies examining the agreement between subjective and objective measures of childhood adversity. Table S6. Egger's test and leave‐one‐out analysis for studies assessing whether subjective and objective measures of childhood adversity independently predict psychopathology. Table S7. Moderators of the association between subjective and objective measures of adverse childhood experiences and psychopathology. Figure S1. PRISMA flow diagram for the study inclusion process. Figure S2. Correlation between subjective and objective measures of bullying victimisation according to study sample size. Figure S3. Forest plot for studies examining the correlation between subjective and objective measures of neighbourhood adversity. Figure S4. Correlation between objective measures of bullying victimisation with psychopathology independent of subjective measures, by study sample size. Figure S5. Forest plot showing the meta‐analytic associations between subjective measures of neighbourhood adversity and psychopathology, independent of objective measures (panel A), and objective measures of neighbourhood adversity and psychopathology, independent of subjective measures (panel B). [file JCPP-64-1185-s001.docx]

**Supporting Information**

**Objective and subjective experiences of childhood adversity: a meta-analysis of their agreement and relationship with psychopathology**

Table of Contents

[Supporting Information 1](#_Toc130395554)

[**Appendix S1.** Search Terms to Identify Eligible Studies 2](#_Toc130395555)

[**Appendix S2.** Variables Extracted 3](#_Toc130395556)

[**Appendix S3.** Converting Unadjusted Correlations to Partial Correlations 6](#_Toc130395557)

[**Table S1.** PRISMA Checklist 7](#_Toc130395558)

[**Table S2.** The Description of Bias Assessment 12](#_Toc130395559)

[**Table S3.** Risk of Bias for All Included Studies (Agreement and Main Meta-Analysis). 16](#_Toc130395560)

[**Table S5.** Egger’s Test and Leave-One-Out Analysis for Studies Examining the Agreement between Subjective and Objective Measures of Childhood Adversity 18](#_Toc130395561)

[**Table S6.** Egger’s Test and Leave-One-Out Analysis for Studies Assessing Whether Subjective and Objective Measures of Childhood Adversity Independently Predict Psychopathology 19](#_Toc130395562)

[**Table S7.** Moderators of the Association Between Subjective and Objective Measures of Adverse Childhood Experiences and Psychopathology 20](#_Toc130395563)

[**Figure S1.** PRISMA Flow Diagram for the Study Inclusion Process 22](#_Toc130395564)

[**Figure S2.** Correlation between Subjective and Objective Measures of Bullying Victimisation, According to Study Sample Size 23](#_Toc130395565)

[**Figure S3**. Forest Plot for Studies Examining the Correlation between Subjective and Objective Measures of Neighbourhood Adversity 24](#_Toc130395566)

[**Figure S4.** Correlation between Objective Measures of Bullying Victimisation with Psychopathology Independent of Subjective Measures, by Study Sample Size 25](#_Toc130395567)

[**Figure S5.** Forest Plot Showing the Meta-Analytic Associations between Subjective Measures of Neighbourhood Adversity and Psychopathology, Independent of Objective Measures (Panel A), and Objective Measures of Neighbourhood Adversity and Psychopathology, Independent of Subjective Measures (Panel B) 26](#_Toc130395568)

[REFERENCES 27](#_Toc130395569)

**Appendix S1.** Search Terms to Identify Eligible Studies

“child* trauma” OR “child* advers*” OR “maltreatment” OR “child* abuse” OR “child* neglect” OR “victim*” OR “bully*” OR “bullie*” OR “neighbourhood viol*” OR “neighborhood viol*” OR “neighbourhood advers*” OR “neighborhood advers*”] AND [“subjective*” OR “perceived” OR “perception*” OR “self-report*”] AND [“objective*” OR “record” OR “agency-notified” OR “peer nom*” OR “peer report*” OR “peer reputation”] AND [“mental health” OR “mental illness” OR “psychopathol*” OR “psychiatric” OR “depress*” OR “anxi*” OR “panic” OR “obsessive compulsive” OR “self inj*” OR “self harm*” OR “suicid*” OR “eating disorder*” OR “schiz*” OR “psychotic” OR “psychosis*” OR “bipolar” OR “attention deficit hyperactivity disorder” OR “conduct” OR “substance abuse” OR “alcohol” OR “drug” OR “cannabis”.

## **Appendix S2.** Variables Extracted

- First author name
- Year of publication
- Cohort name
- Country of study origin
- Percentage female of analytic sample
- Sample size for analysis
- The type of exposure reported by the objective measure
- The type of exposure reported by the subjective measure
- The type of objective measure
- The type of subjective measure
- Observational period for Adverse Childhood Experiences (ACE) reported using objective measure
- Observational period for ACE reported using subjective measure
- The age when subjective measure was obtained
- Variable type for the objective measure
- Variable type for the subjective measure
- The mental health outcome being studied
- The assessment being used to measure mental health outcome
- The informant reporting mental health outcome
- The age when mental health was assessed
- The variable type of psychopathology measure
- Type of effect size reported for the objective measure
- The reported effect size for the association between objective measure and the mental health outcome, controlling for subjective measure
- The standard error reported for the association between objective measure and the mental health outcome, controlling for subjective measure
- The standard deviation reported for the association between objective measure and the mental health outcome, controlling for subjective measure
- The 95% confidence interval reported for the association between objective measure and the mental health outcome, controlling for subjective measure
- The p value reported for the association between objective measure and the mental health outcome, controlling for subjective measure
- Type of effect size reported for the subjective measure
- The reported effect size for the association between subjective measure and the mental health outcome, controlling for objective measure
- The standard error reported for the association between subjective measure and the mental health outcome, controlling for objective measure
- The standard deviation reported for the association between subjective measure and the mental health outcome, controlling for objective measure
- The confidence interval reported for the association between subjective measure and the mental health outcome, controlling for objective measure
- The p value reported for the association between subjective measure and the mental health outcome, controlling for objective measure
- Type of effect size agreement
- Effect size agreement between the subjective and objective measures
- The standard error reported for agreement between the subjective and objective measures
- Number of participants in the total sample that have each measure
- Number of participants who report the objective measure only
- Number of participants who report the subjective measure only
- Number of participants who report the subjective and objective measure only
- Number of participants that have none of the measures reported
- Representativeness of participants classified as exposed to adversity on the objective measure
- Selection of participants classified as not exposed to adversity on the objective measure
- Quality of the subjective assessment of ACE
- Whether the objective and subjective assessments measure exactly the same experiences
- Whether the objective and subjective assessments cover the same time period of exposure
- Demonstration that the mental health outcome was not present before exposure to adversity
- Whether relevant confounding factors controlled for
- Whether subjective measures were administered prior to mental health measures

## **Appendix S3.** Converting Unadjusted Correlations to Partial Correlations

Step 1: Specify correlation matrix with correlations between (i) outcome and the subjective measure, (ii) outcome and the objective measure, and (iii) subjective and the objective measure

Step 2: Generate a covariance matrix from the correlation matrix by specifying standard deviations of the (i) outcome, (ii) objective measure, and (iii) subjective measure*

Step 3: Fit a structural equation model on the covariance matrix, in which the psychopathology outcome is regressed on subjective measures and objective measures of childhood adversity

Step 4: Extract partial correlations and standard errors for the (i) association between the psychopathology outcome and the subjective measure, adjusting for the objective measure, and (ii) association between the psychopathology outcome and the objective measure, adjusting for the subjective measure.

*If the standard deviations were not available, the structural equation model was fitted on the correlation matrix.

## **Table S1.** PRISMA Checklist

| 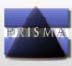**Section and Topic** | **Item #** | **Checklist item** | **Location where item is reported (page)** |
| --- | --- | --- | --- |
| **TITLE** | | |  |
| Title | 1 | Identify the report as a systematic review/meta-analysis | 1 |
| **ABSTRACT** | | |  |
| Abstract | 2 | See the PRISMA 2020 for Abstracts checklist. | 2-3 |
| **INTRODUCTION** | | |  |
| Rationale | 3 | Describe the rationale for the review in the context of existing knowledge. | 4-5 |
| Objectives | 4 | Provide an explicit statement of the objective(s) or question(s) the review addresses. | 5 |
| **METHODS** | | |  |
| Eligibility criteria | 5 | Specify the inclusion and exclusion criteria for the review and how studies were grouped for the syntheses. | 6 & Supp page 2 |
| Information sources | 6 | Specify all databases, registers, websites, organisations, reference lists and other sources searched or consulted to identify studies. Specify the date when each source was last searched or consulted. | 7 |
| Search strategy | 7 | Present the full search strategies for all databases, registers and websites, including any filters and limits used. | 7 & Supp page 2 |
| Selection process | 8 | Specify the methods used to decide whether a study met the inclusion criteria of the review, including how many reviewers screened each record and each report retrieved, whether they worked independently, and if applicable, details of automation tools used in the process. | 6-7 |
| Data collection process | 9 | Specify the methods used to collect data from reports, including how many reviewers collected data from each report, whether they worked independently, any processes for obtaining or confirming data from study investigators, and if applicable, details of automation tools used in the process. | 7-8 |
| Data items | 10a | List and define all outcomes for which data were sought. Specify whether all results that were compatible with each outcome domain in each study were sought (e.g. for all measures, time points, analyses), and if not, the methods used to decide which results to collect. | Supp pages 3-5 |
|  | 10b | List and define all other variables for which data were sought (e.g. participant and intervention characteristics, funding sources). Describe any assumptions made about any missing or unclear information. | Supp pages 3-5 |
| Study risk of bias assessment | 11 | Specify the methods used to assess risk of bias in the included studies, including details of the tool(s) used, how many reviewers assessed each study and whether they worked independently, and if applicable, details of automation tools used in the process. | 9, 12 & Supp pages 12-14 |
| Effect measures | 12 | Specify for each outcome the effect measure(s) (e.g. risk ratio, mean difference) used in the synthesis or presentation of results. | 10-11 |
| Synthesis methods | 13a | Describe the processes used to decide which studies were eligible for each synthesis (e.g. tabulating the study intervention characteristics and comparing against the planned groups for each synthesis (item #5)). | 7-8 |
|  | 13b | Describe any methods required to prepare the data for presentation or synthesis, such as handling of missing summary statistics, or data conversions. | 8-9 |
|  | 13c | Describe any methods used to tabulate or visually display results of individual studies and syntheses. | 8-9 |
|  | 13d | Describe any methods used to synthesize results and provide a rationale for the choice(s). If meta-analysis was performed, describe the model(s), method(s) to identify the presence and extent of statistical heterogeneity, and software package(s) used. | 8-9 |
|  | 13e | Describe any methods used to explore possible causes of heterogeneity among study results (e.g. subgroup analysis, meta-regression). | 9 |
|  | 13f | Describe any sensitivity analyses conducted to assess robustness of the synthesized results. | 10 |
| Reporting bias assessment | 14 | Describe any methods used to assess risk of bias due to missing results in a synthesis (arising from reporting biases). | 10 |
| Certainty assessment | 15 | Describe any methods used to assess certainty (or confidence) in the body of evidence for an outcome. | NA |
| **RESULTS** | | |  |
| Study selection | 16a | Describe the results of the search and selection process, from the number of records identified in the search to the number of studies included in the review, ideally using a flow diagram. | 10-11 & Supp Figure 1 |
|  | 16b | Cite studies that might appear to meet the inclusion criteria, but which were excluded, and explain why they were excluded. | Table 1 |
| Study characteristics | 17 | Cite each included study and present its characteristics. | Table 1 |
| Risk of bias in studies | 18 | Present assessments of risk of bias for each included study. | Supp page 15 |
| Results of individual studies | 19 | For all outcomes, present, for each study: (a) summary statistics for each group (where appropriate) and (b) an effect estimate and its precision (e.g. confidence/credible interval), ideally using structured tables or plots. | Table 1, Table 2 |
| Results of syntheses | 20a | For each synthesis, briefly summarise the characteristics and risk of bias among contributing studies. | Supp page 12-15 |
|  | 20b | Present results of all statistical syntheses conducted. If meta-analysis was done, present for each the summary estimate and its precision (e.g. confidence/credible interval) and measures of statistical heterogeneity. If comparing groups, describe the direction of the effect. | 10-15 |
|  | 20c | Present results of all investigations of possible causes of heterogeneity among study results. | 10-15 |
|  | 20d | Present results of all sensitivity analyses conducted to assess the robustness of the synthesized results. | NA |
| Reporting biases | 21 | Present assessments of risk of bias due to missing results (arising from reporting biases) for each synthesis assessed. | NA |
| Certainty of evidence | 22 | Present assessments of certainty (or confidence) in the body of evidence for each outcome assessed. | NA |
| **DISCUSSION** | | |  |
| Discussion | 23a | Provide a general interpretation of the results in the context of other evidence. | 15-20 |
|  | 23b | Discuss any limitations of the evidence included in the review. | 19 |
|  | 23c | Discuss any limitations of the review processes used. | 19 |
|  | 23d | Discuss implications of the results for practice, policy, and future research. | 19-20 |
| **OTHER INFORMATION** | | |  |
| Registration and protocol | 24a | Provide registration information for the review, including register name and registration number, or state that the review was not registered. | 6 |
|  | 24b | Indicate where the review protocol can be accessed, or state that a protocol was not prepared. | 6 |
|  | 24c | Describe and explain any amendments to information provided at registration or in the protocol. | 7 |
| Support | 25 | Describe sources of financial or non-financial support for the review, and the role of the funders or sponsors in the review. | 21 |
| Competing interests | 26 | Declare any competing interests of review authors. | 21 |
| Availability of data, code and other materials | 27 | Report which of the following are publicly available and where they can be found: template data collection forms; data extracted from included studies; data used for all analyses; analytic code; any other materials used in the review. | 10 |

## **Table S2.** The Description of Bias Assessment

The following table corresponds to the description of the information that was extracted in order to assess the bias of included studies. We adapted the Newcastle-Ottowa scale (Wells et al, 2000) with relevant items to assess the quality of studies in examining the associations between subjective and objective measures of childhood adversity with psychopathology. For example, we added items on whether the subjective and objective measures capture exactly the same experience and cover the same time period, and whether psychopathology was assessed longitudinally or cross-sectionally. These additional questions resulted in a 9-point scale ranging from 0 (indicating very high bias) to 8 (indicating very low bias).

| **Bias assessed** | **Description** | **Assignment of score** |
| --- | --- | --- |
| **Exposed group is representative** | *Representativeness of participants classified as exposed to adversity on the objective measure* | A score of 0 or 1 (as outlined below) depending on whether participants classified as exposed to adversity on the objective measure are:  a) truly representative of the average cohort in the community (1)  b) somewhat representative of the average cohort in the community (1)  c) selected group: eg. children selected because of exposure to maltreatment/other adversity (0)  d) no description of the derivation of the exposed group (0) |
| **Selection of controls** | *Selection of participants classified as not exposed to adversity on the objective measure* | A score of 0 or 1 depending on whether participants classified as unexposed on the objective measure are:   1. drawn from the same community as those classified as “exposed” on the objective measure or matched to ensure comparability (1). 2. drawn from a different source (0). 3. No description of the derivation of the unexposed group (0). |
| **Quality of subjective measure** | *Quality of the subjective assessment of ACE* | A score of 0 or 1 depending on whether the self-report assessment of adversity was based on a:   1. Interview or questionnaire tested for validity and reliability (1). 2. non-validated self-report questionnaire/interview or no description (0). |
| **Comparison of ACE measures** | *Whether the objective and subjective assessments measure exactly the same experiences.* | A score of 0 or 1 (as outlined below):   1. The subjective and objective measures assess exactly the same ACE experiences i.e., child maltreatment, bullying victimisation (1). 2. The subjective and objective measures do not assess different ACE experiences i.e., objective = neighbourhood crime records, subjective = neighbourhood disorder (0). 3. It is unclear whether the subjective and objective measures assess the same thing (0). |
| **Comparison of exposure time** | *Whether the objective and subjective assessments cover the same time period of exposure* | A score of 0 or 1 (as outlined below:   1. The time-period of exposure to adversity covered by the objective and subjective measures was exactly the same (e.g., court records and self-reports measured adversity between birth and age 12) (1) 2. The time-period of exposure to adversity covered by the objective and subjective measures was different (e.g., court records assessed adversity between birth and age 12; self-reports assessed adversity between birth and age 18) (0) |
| **Control for pre-existing mental health** | *Demonstration that the mental health outcome was not present before exposure to adversity* | A score of 0 or 1 (as outlined below:   1. Pre-existing mental health outcomes were controlled for in the analysis (or participants with pre-existing mental health problems were removed) (1) 2. Mental health outcome(s) was not controlled for (0). |
| **Confounding** | *Whether relevant confounding factors controlled for.* | A score of 0 or 1 (as outlined below:   1. The study controlled for any of the following confounders:  - socioeconomic status - parental education - family income - other adversities (e.g., poverty, bullying, maltreatment, victimisation) - genetic risk for mental health problems (family history of psychopathology, polygenic score) (1).  1. Did not control for any of the above (0). |
| **Longitudinal vs. cross-sectional study** | *Whether* *subjective measures were administered prior to mental health measures* | A score of 0 or 1 (as outlined below):   1. Subjective measures (e.g., self-reports) of adversity were collected prior to mental health outcomes (e.g., if self-reports were measured at age 8 and mental health was measured at age 10) (1). 2. Subjective measures were administered at the same time or after the assessment of mental health measures (0). 3. It is unclear when subjective measures were administered (0). |

|  | Exposed group is representative | Selection of controls | Quality of subjective measure | Comparison of ACE measures | Comparison of exposure time | Control for mental health | Confounding | Longitudinal | Total risk of bias score* |
| --- | --- | --- | --- | --- | --- | --- | --- | --- | --- |
| Bouman et al (2012) | 1 | 1 | 1 | 1 | 1 | 0 | 0 | 0 | 5 |
| Cho & Jackson (2016) | 0 | 1 | 1 | 1 | 1 | 0 | 0 | 0 | 4 |
| Danese & Widom (2020) | 0 | 1 | 1 | 1 | 1 | 0 | 1 | 0 | 5 |
| De Los Reyes & Prinstein (2004) | 1 | 1 | 1 | 1 | 1 | 0 | 0 | 0 | 5 |
| Everson et al (2008) | 0 | 0 | 0 | 1 | 1 | 0 | 1 | 0 | 3 |
| Flanagan, Erath & Bierman (2008) | 1 | 0 | 1 | 1 | 1 | 0 | 0 | 0 | 4 |
| Graham, Bellmore & Juvonen (2003) | 1 | 1 | 0 | 1 | 1 | 0 | 0 | 0 | 4 |
| Graham & Juvonen (1998) | 1 | 1 | 1 | 1 | 1 | 0 | 0 | 0 | 5 |
| Goldman-Mellor, Margerison-Zilko, Allen & Cerda (2016) | 1 | 1 | 0 | 0 | 1 | 0 | 1 | 0 | 4 |
| Gromann, Goossens, Olthof, Pronk & Krabbendam (2013) | 1 | 1 | 1 | 1 | 0 | 0 | 0 | 0 | 4 |
| Havlicek & Courtney (2016) | 0 | 1 | 1 | 1 | 1 | 0 | 0 | 0 | 4 |
| Kochel, Bagwell, Ladd & Rudolph (2017) | 1 | 1 | 0 | 1 | 1 | 0 | 0 | 1 | 5 |
| McClain, Younginer & Elledge (2020) | 1 | 1 | 1 | 0 | 1 | 1 | 0 | 1 | 6 |
| McGee, Wolfe, Yuen, Wilson & Carnochan (1995) | 0 | 1 | 1 | 1 | 1 | 0 | 0 | 0 | 4 |
| Mulder, Hutteman & van Aken (2017) | 1 | 1 | 1 | 1 | 1 | 0 | 0 | 1 | 6 |
| Negriff, Schneiderman & Trickett (2017) | 0 | 1 | 1 | 1 | 1 | 0 | 0 | 0 | 4 |
| Newbury et al (2017) | 1 | 1 | 0 | 0 | 0 | 1 | 1 | 0 | 4 |
| Rigby & Slee (1999) | 1 | 1 | 1 | 1 | 1 | 0 | 0 | 0 | 5 |
| Sierau et al (2017) | 0 | 0 | 1 | 1 | 1 | 0 | 0 | 0 | 3 |
| Smith, Ireland, Thornberry & Elwyn (2008) | 1 | 1 | 0 | 1 | 0 | 1 | 1 | 0 | 5 |
| White, English, Thompson & Roberts (2016) | 0 | 0 | 1 | 1 | 1 | 0 | 0 | 1 | 4 |
| Zimmer-Gembeck & Pronk (2012) | 1 | 1 | 0 | 1 | 1 | 0 | 0 | 0 | 4 |

## **Table S3.** Risk of Bias for All Included Studies (Agreement and Main Meta-Analysis).

*Risk of bias total scores ranged from 2 to 6 out of 8, with 0 being the highest risk of bias.

**Table S4.** Formulae for Conversion to Correlation

| **Raw effect size type** | **Formula for conversion to *r*** | **Reference** |
| --- | --- | --- |
| **Cohen’s *d*** | $r= \frac{d}{\sqrt{d^{2}+a}}$ | Borenstein, Hedges, Higgins & Rothstein (2021) |
| **Log odds ratio** | Step 1:  $d=LogOddsRatio x \frac{\sqrt{3}}{\pi}$  $\text{Step 2:}\text{ }$  $r\boldsymbol{=}\frac{d}{\sqrt{d^{2}+a}}$ | Borenstein, Hedges, Higgins & Rothstein (2021) |
| **Risk Ratio** (RR) | Step 1:  $LogOddsRatio=\frac{\log\left( 1-p \right)*RR}{1-RR*p}$  Step 2:  $d=LogOddsRatio \times\frac{\surd3}{\pi}$  Step 3:  $r\boldsymbol{=}\frac{d}{\sqrt{d^{2}+a}}$ | Grant (2014) |
| **Unstandardised beta** $\boldsymbol{(}\beta)$ | $r =\beta\left( \frac{sd exposure}{sd outcome} \right)$ | Cross validated (2022) Accessed: 22nd June 2022 |

RR= Risk Ratio; *p*= the control event rate (prevalence in unexposed population)*; d=* Cohen’s d*; se=* standard error; $a$ is the correction factor for cases where $n_{1}\neq n_{2}.$ If $n_{1}$ and $n_{2}$ are not precisely known, then $a=4$***;*** OR= Odds Ratio; CI= Confidence Interval; $\beta= Unstandardised Coefficient; r = Correlation Coefficient;$ sd= Standard Deviation; sd exposure = Standard Deviation of the Exposure Variable Reported; sd outcome = Standard Deviation of the Outcome Variable Reported.

## **Table S5.** Egger’s Test and Leave-One-Out Analysis for Studies Examining the Agreement between Subjective and Objective Measures of Childhood Adversity

| **Agreement meta-analysis** | **Egger’s Test** | **Leave-one-out analysis** | | **Meta-analytic effect size for comparison** |
| --- | --- | --- | --- | --- |
|  | Q_mod (*p*) | **Smallest effect size**  *r* (95% CI) | **Largest effect size**  *r* (95% CI) |  |
| **Maltreatment**  (meta-analysis of correlations) | 2.59 (0.11) | 0.29 (0.22-0.35) (McGee, Wolfe, Yuen, Wilson & Carnochan, 1995)* | 0.34 (0.25-0.43)  (White, English, Thompson & Roberts, 2016) | 0.32 (0.23-0.41) |
| **Maltreatment**  (meta-analysis of kappas) | 0.46 (0.50) | 0.13 (0.09-0.17)  (McGee, Wolfe, Yuen, Wilson & Carnochan, 1995) | 0.17 (0.10-0.24)  (Cho & Jackson, 2016) | 0.16 (0.10-0.22) |
| **Bullying**  (meta-analysis of correlations) | 7.80 (0.0052) | 0.32 (0.26-0.38)  (Gromann, Goossens, Olthof, Pronk & Krabbendam, 2013) | 0.36 (0.28-0.44)  (De Los Reyes & Prinstein, 2004) | 0.35 (0.27-0.42) |

*The study reference removed.

## **Table S6.** Egger’s Test and Leave-One-Out Analysis for Studies Assessing Whether Subjective and Objective Measures of Childhood Adversity Independently Predict Psychopathology

| **Main meta-analysis** | **Egger’s Test** | | **Leave-one-out analysis** | | **Meta-analytic effect size for comparison**  *r* (95% CI) |
| --- | --- | --- | --- | --- | --- |
|  | Q_mod (*p*) | | **Smallest effect size**  *r* (95% CI) | **Largest effect size**  *r* (95% CI) |  |
| **Maltreatment** (subjective measure) | 0.02 (0.89) | | 0.14(0.07-0.20)  (Cho & Jackson, 2016)***** | 0.18(0.13-0.23)  White, English, Thompson & Roberts (2016) | 0.16(0.09-0.22) |
| **Maltreatment** (objective measure) | 0.01 (0.93) | 0.02(-0.02-0.07)  Cho & Jackson, 2016) | | 0.08(-0.00-0.15)  Danese & Widom (2020) | 0.06(-0.02-0.13) |
| **Bullying**  (subjective measure) | 1.54 (0.21) | | 0.11(0.07-0.14)  Bouman et al (2012) | 0.13(0.09-0.17) Kochel, Bagwell, Ladd & Rudolph (2017) | 0.12(0.08-0.17) |
| **Bullying**  (objective measure) | 4.81 (0.03) | | 0.02(-0.02-0.06)  Mulder, Hutteman & van Aken (2017) | 0.04(-0.00-0.09)  McClain, Younginer & Elledge (2020) | 0.03(-0.01-0.08) |

*The study reference removed.

**Table S7.** Moderators of the Association Between Subjective and Objective Measures of Adverse Childhood Experiences and Psychopathology

| **Moderators by adversity type** | **No of studies reporting each outcome** | **No. of effect sizes** | **Effect Size Estimate,**  ***r* (95% CI)** | **QM** | ***QM P* value** |
| --- | --- | --- | --- | --- | --- |
| **Childhood maltreatment** | | | | | |
| **Subjective measure** | | | | | |
| *Informant for psychopathology* | | | | 1.93 | 0.16 |
| Self-report | 5 | 88 | 0.14 (0.07-0.20) |  |  |
| Other | 1* | 6 | 0.26 (0.10-0.41) |  |  |
| *Study type* | | | | 7.15 | **0.0075** |
| Cross-sectional | 5 | 82 | 0.18 (0.13-0.23) |  |  |
| Longitudinal | 1* | 12 | 0.03 (-0.07-0.14) |  |  |
| *Type of psychopathology^a^* | | | | 0.09 | 0.77 |
| Externalising problems | 4 | 36 | 0.15 (0.06-0.24) |  |  |
| Internalising problems | 5 | 45 | 0.16 (0.07-0.24) |  |  |
| *Study quality* | 6 | 94 | 0.02 (-0.07-0.12) | 0.28 | 0.60 |
| *Sex (percent female)* | 6 | 94 | -0.02 (-0.03-0.01) | 30.34 | **<0.0001** |
| **Objective measure** | | | | | |
| *Informant for psychopathology* | | | | 9.62 | **0.0019** |
| Self-report | 5 | 88 | 0.02 (-0.02-0.07) |  |  |
| Other | 1* | 6 | 0.21 (0.10-0.32) |  |  |
| *Study type* | | | | 0.005 | 0.95 |
| Cross-sectional | 5 | 82 | 0.05 (-0.03-0.13) |  |  |
| Longitudinal | 1* | 12 | 0.01 (-0.16-0.17) |  |  |
| *Type of psychopathology^a^* | | | | 0.09 | 0.76 |
| Externalising problems | 4 | 36 | 0.05 (-0.03-0.14) |  |  |
| Internalising problems | 5 | 45 | 0.06 (-0.02-0.14) |  |  |
| *Study quality* | 6 | 94 | -0.03 (-0.12-0.05) | 0.62 | 0.43 |
| *Sex (percent female)* | 6 | 94 | -0.01 (-0.03-0.01) | 1.34 | 0.25 |
| **Bullying victimisation** | | | | | |
| **Subjective measure** | | | | | |
| *Informant for psychopathology* | | | | 20.37 | **<0.0001** |
| Self-report | 8 | 30 | 0.15 (0.11-0.20) |  |  |
| Other | 3 | 15 | 0.03 (-0.04-0.09) |  |  |
| Study type | | | | 1.58 | 0.21 |
| Cross-sectional | 9 | 31 | 0.13 (0.08-0.17) |  |  |
| Longitudinal | 3 | 14 | 0.09 (0.03-0.16) |  |  |
| *Type of psychopathology* | | | | 0.21 | 0.64 |
| Externalising problems | 1* | 1* | 0.08 (-0.07-0.24) |  |  |
| Internalising problems | 8 | 43 | 0.12 (0.07-0.17) |  |  |
| *Study quality* | 9 | 45 | -0.00 (-0.05-0.04) | 0.01 | 0.92 |
| *Sex (percent female)* | 9 | 45 | 0.00 (-0.00-0.00) | 0.14 | 0.71 |
| **Objective measure** | | | | | |
| *Informant for psychopathology* | | | | 3.30 | 0.07 |
| Self-report | 8 | 30 | 0.02 (-0.04-0.07) |  |  |
| Other | 3 | 15 | 0.08 (0.01-0.15) |  |  |
| *Study type* | | | | 0.06 | 0.81 |
| Cross-sectional | 9 | 31 | 0.03 (-0.01-0.08) |  |  |
| Longitudinal | 3 | 14 | 0.03 (-0.04-0.09) |  |  |
| *Type of psychopathology* | | | | 145.01 | **<0.0001** |
| Externalising problems | 1* | 1* | 0.47 (0.40-0.53) |  |  |
| Internalising problems | 8 | 43 | 0.02 (-0.02-0.07) |  |  |
| *Study quality* | 9 | 45 | -0.00 (-0.05-0.04) | 0.03 | 0.87 |
| *Sex (percent female)* | 9 | 45 | -0.00 (-0.00-0.00) | 1.11 | 0.29 |

* These estimates are based on only a single study and/or single effect size and are presented for transparency, but should be interpreted with caution.

*^a^*Everson et al (2008) not included in the analysis for moderation by mental health outcome as “psychological adjustment” outcome reported was a positive indicator of wellbeing rather than mental ill health.

## **Figure S1.** PRISMA Flow Diagram for the Study Inclusion Process

Records identified through an initial review process of MEDLINE, PsychINFO, and Embase

*(n = 4,417)*

Additional records identified through other sources

*(n = 4)*

Duplicates removed

*(n = 27)*

Records after duplicates removed

*(n = 4,394)*

Total records identified

*(n = 4,421)*

Records excluded based on title or abstract

*(n = 4,326)*

Records excluded based on full text

*(n = 42)*

Full-text articles assessed for eligibility

*(n = 68)*

Records excluded as additional information required were not provided by authors

(*n* = *4*)

Full-text identified as meeting eligibility criteria

*(n = 26)*

Studies included in the meta-analysis on agreement

*(n = 22)*

Records excluded as did not contain effect sizes that could be extracted or calculated from the data available

*(n = 5)*

Studies included in the meta-analysis on psychopathology

*(n = 17)*

## **Figure S2.** Correlation between Subjective and Objective Measures of Bullying Victimisation, According to Study Sample Size


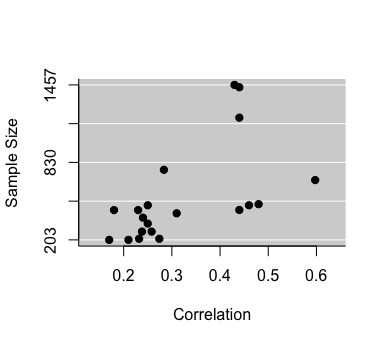


## **Figure S3**. Forest Plot for Studies Examining the Correlation between Subjective and Objective Measures of Neighbourhood Adversity

## **Figure S4.** Correlation between Objective Measures of Bullying Victimisation with Psychopathology Independent of Subjective Measures, by Study Sample Size

**
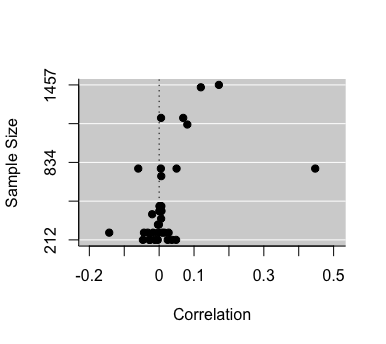
**

## **Figure S5.** Forest Plot Showing the Meta-Analytic Associations between Subjective Measures of Neighbourhood Adversity and Psychopathology, Independent of Objective Measures (Panel A), and Objective Measures of Neighbourhood Adversity and Psychopathology, Independent of Subjective Measures (Panel B)

### **REFERENCES**

Borenstein, M., Hedges, L. V., Higgins, J. P. T., & Rothstein, H. R. (2021). *Introduction to Meta-Analysis*. John Wiley & Sons.

Bouman, T., van der Meulen, M., Goossens, F. A., Olthof, T., Vermande, M. M., & Aleva, E. A. (2012). Peer and self-reports of victimization and bullying: Their differential association with internalizing problems and social adjustment. *Journal of School Psychology*, *50*(6), 759–774.

Cho, B., & Jackson, Y. (2016). Self-reported and case file maltreatment: Relations to psychosocial outcomes for youth in foster care. *Children and Youth Services Review*, *69*, 241–247.

*Cross Validated* (2022). Available at: https://stats.stackexchange.com/questions/451358/calculating-the-standard-errors-of-the-standardized-regression-coefficients-from. (Accessed: 22nd June 2022)

Danese, A., & Widom, C. S. (2020). Objective and subjective experiences of child maltreatment and their relationships with psychopathology. *Nature Human Behaviour*, *4*(8), 811–818.

De Los Reyes, A., & Prinstein, M. J. (2004). Applying Depression-Distortion Hypotheses to the Assessment of Peer Victimization in Adolescents. *Journal of Clinical Child & Adolescent Psychology*, *33*(2), 325–335.

Everson, M. D., Smith, J. B., Hussey, J. M., English, D., Litrownik, A. J., Dubowitz, H., Thompson, R., Dawes Knight, E., & Runyan, D. K. (2008). Concordance Between Adolescent Reports of Childhood Abuse and Child Protective Service Determinations in an At-Risk Sample of Young Adolescents. *Child Maltreatment*, *13*(1), 14–26.

Flanagan, K. S., Erath, S. A., & Bierman, K. L. (2008). Unique Associations Between Peer Relations and Social Anxiety in Early Adolescence. *Journal of Clinical Child & Adolescent Psychology*, *37*(4), 759–769.

Goldman-Mellor, S., Margerison-Zilko, C., Allen, K., & Cerda, M. (2016). Perceived and Objectively-Measured Neighborhood Violence and Adolescent Psychological Distress. *Journal of Urban Health*, *93*(5), 758–769.

Graham, S., Bellmore, A., & Juvonen, J. (2003). Peer Victimization in Middle School: When Self-and Peer Views Diverge. *Journal of Applied School Psychology*, *19*(2), 117–137.

Graham, S., & Juvonen, J. (1998). Self-blame and peer victimization in middle school: an attributional analysis. *Developmental psychology*, *34*(3), 587.

Grant, R. L. (2014). Converting an odds ratio to a range of plausible relative risks for better communication of research findings. *Bmj*, *348*.

Gromann, P. M., Goossens, F. A., Olthof, T., Pronk, J., & Krabbendam, L. (2013). Self-perception but not peer reputation of bullying victimization is associated with non-clinical psychotic experiences in adolescents. *Psychological Medicine*, *43*(4), 781–787.

Havlicek, J., & Courtney, M. E. (2016). Maltreatment histories of aging out foster youth: A comparison of official investigated reports and self-reports of maltreatment prior to and during out-of-home care. *Child Abuse & Neglect*, *52*, 110–122.

Kochel, K. P., Bagwell, C. L., Ladd, G. W., & Rudolph, K. D. (2017). Do positive peer relations mitigate transactions between depressive symptoms and peer victimization in adolescence? *Journal of Applied Developmental Psychology*, *51*, 44–54.

McClain, C. M., Younginer, S. T., & Elledge, L. C. (2020). Social Risk and Internalizing Distress in Middle Childhood: The Moderating Role of Emotion Regulation Processes. *Journal of Child and Family Studies*, *29*(1), 167–181.

McGee, R. A., Wolfe, D. A., Yuen, S. A., Wilson, S. K., & Carnochan, J. (1995). The measurement of maltreatment: A comparison of approaches. *Child Abuse & Neglect*, *19*(2), 233–249.

Mulder, S. F., Hutteman, R., & van Aken, M. A. G. (2017). Predictive effects of social anxiety on increases in future peer victimization for a community sample of middle-school youth. *International Journal of Behavioral Development*, *41*(5), 588–596.

Negriff, S., Schneiderman, J. U., & Trickett, P. K. (2017). Concordance Between Self-Reported Childhood Maltreatment Versus Case Record Reviews for Child Welfare–Affiliated Adolescents: Prevalence Rates and Associations With Outcomes. *Child Maltreatment*, *22*(1), 34–44.

Newbury, J. B., Arseneault, L., Caspi, A., Moffitt, T. E., Odgers, C. L., Baldwin, J. R., Zavos, H. M. S., & Fisher, H. L. (2017). In the eye of the beholder: Perceptions of neighborhood adversity and psychotic experiences in adolescence. *Development and Psychopathology*, *29*(5), 1823–1837.

Rigby, K., & Slee, P. (1999). Suicidal ideation among adolescent school children, involvement in bully—victim problems, and perceived social support. *Suicide and Life‐Threatening Behavior*, *29*(2), 119-130.

Sierau, S., Brand, T., Manly, J. T., Schlesier-Michel, A., Klein, A. M., Andreas, A., Garzón, L. Q., Keil, J., Binser, M. J., von Klitzing, K., & White, L. O. (2017). A Multisource Approach to Assessing Child Maltreatment From Records, Caregivers, and Children. *Child Maltreatment*, *22*(1), 45–57.

Smith, C. A., Ireland, T. O., Thornberry, T. P., & Elwyn, L. (2008). Childhood maltreatment and antisocial behavior: Comparison of self‐reported and substantiated maltreatment. *American Journal of Orthopsychiatry*, *78*(2), 173-186.

Wells, G. A., Shea, B., O’Connell, D., Peterson, J., Welch, V., Losos, M., & Tugwell, P. (2000). The Newcastle-Ottawa Scale (NOS) for assessing the quality of nonrandomised studies in meta-analyses.

White, C. R., English, D., Thompson, R., & Roberts, Y. H. (2016). Youth self-report of emotional maltreatment: Concordance with official reports and relation to outcomes. *Children and Youth Services Review*, *62*, 111–121.

Zimmer-Gembeck, M. J., & Pronk, R. E. (2012). Relation of Depression and Anxiety to Self- and Peer-Reported Relational Aggression: Depression, Anxiety, and Relational Aggression. *Aggressive Behavior*, *38*(1), 16–30.
